# Impact of clinical parameters and systemic inflammatory status on epidermal growth factor receptor-mutant non-small cell lung cancer patients readministration with epidermal growth factor receptor tyrosine kinase inhibitors

**DOI:** 10.1186/s12885-016-2917-6

**Published:** 2016-11-08

**Authors:** Yu-Mu Chen, Chien-Hao Lai, Kun-Ming Rau, Cheng-Hua Huang, Huang-Chih Chang, Tung-Ying Chao, Chia-Cheng Tseng, Wen-Feng Fang, Yu-Hsiu Chung, Yi-Hsi Wang, Mao-Chang Su, Kuo-Tung Huang, Shih-Feng Liu, Hung-Chen Chen, Ya-Chun Chang, Yu-Ping Chang, Chin-Chou Wang, Meng-Chih Lin

**Affiliations:** 1 Division of Pulmonary and Critical Care Medicine, Department of Internal Medicine, Chang Gung Memorial Hospital-Kaohsiung Medical Center, Chang Gung University College of Medicine, No. 123, Ta-Pei Road, Niao-Sung District, Kaohsiung City, Taiwan; 2 Division of Hematology-Oncology, Department of Internal Medicine, Kaohsiung Chang Gung Memorial Hospital and Chang Gung University College of Medicine, Kaohsiung, Taiwan; 3 Department of Respiratory Care, Chang Gung University of Science and Technology, Chiayi Campus, Chiayi, Taiwan

**Keywords:** Neutrophil-to-lymphocyte ratio, Lymphocyte-to-monocyte ratio, Readministration, Non-small cell lung cancer, Epidermal growth factor receptor, Tyrosine kinase inhibitor

## Abstract

**Background:**

Epidermal growth factor receptor (EGFR)-tyrosine kinase inhibitor (TKI) readministration to lung cancer patients is common owing to the few options available. Impact of clinical factors on prognosis of EGFR-mutant non-small cell lung cancer (NSCLC) patients receiving EGFR-TKI readministration after first-line EGFR-TKI failure and a period of TKI holiday remains unclear. Through this retrospective study, we aimed to understand the impact of clinical factors in such patients.

**Methods:**

Of 1386 cases diagnosed between December 2010 and December 2013, 80 EGFR-mutant NSCLC patients who were readministered TKIs after failure of first-line TKIs and intercalated with at least one cycle of cytotoxic agent were included. We evaluated clinical factors that may influence prognosis of TKI readministration as well as systemic inflammatory status in terms of neutrophil-to-lymphocyte ratio (NLR) and lymphocyte-to-monocyte ratio (LMR).

Baseline NLR and LMR were estimated at the beginning of TKI readministration and trends of NLR and LMR were change amount from patients receiving first-Line TKIs to TKIs readministration.

**Results:**

Median survival time since TKI readministration was 7.0 months. In the univariable analysis, progression free survival (PFS) of first-line TKIs, baseline NLR and LMR, and trend of LMR were prognostic factors in patients receiving TKIs readministration. In the multivariate analysis, only PFS of first-line TKIs (*p* < 0.001), baseline NLR (*p* = 0.037), and trend of LMR (*p* = 0.004) were prognostic factors.

**Conclusion:**

Longer PFS of first-line TKIs, low baseline NLR, and high trend of LMR were good prognostic factors in EGFR-mutant NSCLC patients receiving TKI readministration.

**Electronic supplementary material:**

The online version of this article (doi:10.1186/s12885-016-2917-6) contains supplementary material, which is available to authorized users.

## Background

Lung cancer is the leading cause of cancer-related deaths in Taiwan and worldwide [1, 2]. Although epidermal growth factor receptor (EGFR)-tyrosine kinase inhibitors (TKIs) are administered as standard first-line regimen for advanced EGFR-mutant non-small cell lung cancer (NSCLC) [3–5], the salvage treatment for cases with acquired resistance to EGFR-TKIs remains unclear. Owing to several barriers including difficulty of tumor re-biopsy, absence of EGFR T790m mutation or programmed death-ligand 1 expression, and high expenses, some patients do not have an opportunity to receive novel agents such as 3^rd^ generation TKI [6] or immunotherapies [7, 8].

In patients with acquired resistance to EGFR-TKIs, readministration of first or second generation EGFR-TKIs has been proved to effectively increase patients’ survival time [9–11]. In non-selective patients, EGFR-TKI readministration has only modest efficacy with a progression free survival (PFS) of 2–4 months [10, 12]. However, in optimal selected patients, patients could have a PFS of more than 6 months [10]. Although several good prognostic factors for patients receiving TKI readministration have been reported, such as EGFR-TKI free holidays, better Eastern Cooperative Oncology Group performance status, and benefit from prior EGFR-TKI therapy [10–12], little is known about the correlation between systemic inflammatory markers and TKI readministration efficacies. In previous studies, several systemic inflammatory markers were found to be prognostic factors in lung cancer patients. NSCLC patients with higher blood neutrophil-to-lymphocyte ratio (NLR) had poor prognosis when treated with a combination of bevacizumab and cytotoxic agents [13]; those with higher lymphocyte-to-monocyte ratio (LMR) had better prognosis in EGFR-mutant NSCLC patients receiving first-line EGFR-TKIs [14].

Based on these aforementioned reasons we performed a retrospective study to understand the impact of clinical factors including NLR and LMR on EGFR-mutant NSCLC patients receiving TKI readministration. To decrease the impact of confounding factors, we only included EGFR-mutant NSCLC patients receiving TKI readministration as third or later line therapies after failure of first-line EGFR-TKIs and at least one cycle of intercalated chemotherapy.

## Methods

### Patients and clinical characteristics

We conducted a retrospective study between December 2010 and December 2013 at Kaohsiung Chang Gung Memorial Hospital in Taiwan. Patients were followed-up until November 2015. Adult patients aged ≥18 years with histologically or cytologically confirmed stage IIIB or IV NSCLC who had been treated with first line EGFR-TKIs and received TKIs readministration were included. Patients who had received a second TKI without intercalating with at least one cycle of cytotoxic chemotherapies were excluded.

Baseline assessments including clinical parameters, hematological variables, biochemistry, chest radiography, and chest computed tomography were performed within 4 weeks of initiation of TKI readministration.

Clinical parameters included length of TKI holiday and PFS of study patients receiving first line EGFR-TKIs. Data regarding hematological parameters were collected within 4 weeks of the initiation of first-line TKI therapy and also TKI readministration including neutrophil, lymphocyte, and monocyte counts. NLR was obtained by dividing the neutrophil count by the lymphocyte count, and LMR was obtained by dividing the lymphocyte count by the monocyte count. Baseline NLR and LMR were estimated at the beginning of TKI readministration and trends of NLR and LMR were obtained by dividing the data estimated at the beginning of TKI readministration with the data estimated at the beginning of first-line TKIs.

This study was approved by the Institutional Review Board of Kaohsiung Chang Gung Memorial Hospital. The need for informed consent was waived.

### EGFR mutation testing

Tumor specimens were obtained by bronchoscopy CT-guided biopsy, pleural effusion cytology, or surgical procedures. The *EGFR* mutational analyses was performed using SCORPIONS and ARMS polymerase chain reaction using fragments amplified from genomic DNA extracted from paraffin-embedded tissues (QIAGEN EGFR RGQ PCR KIT). Exon 19 deletion and L858R mutations were defined as common mutations. Other mutations or compound mutations were defined as uncommon mutations.

### Evaluation of response to EGFR-TKI readministration

Patients underwent routine chest radiography every 2–4 weeks and chest computed tomography every 2–3 months to evaluate tumor responses. PFS was defined as the time between the first day of EGFR-TKI administration and disease progression, death before documented progression, or the last visit during the follow-up period. Disease progression was determined by the clinician according to the Response Evaluation Criteria in Solid Tumors criteria 1.1 [15]. The endpoint was overall survival (OS), which was defined as the first day of EGFR-TKI readministration until death, or the last visit during the follow-up period.

### Statistical analyses

Statistical analyses were performed using MedCalc (version 14.10.2). Receiver operating characteristic (ROC) curves with binary variable of OS longer or shorter than 7.0 months since readministration and Youden’s index were used to determine the best cut-off value for baseline values of and trends of NLR LMR as a prognostic factors. OS analyses were performed using the Kaplan-Meier method and the log-rank test. Cox proportional hazards regression test were used to evaluate independent factors. *P* value < 0.05 was considered significant in statistical tests.

## Results

### Patient characteristics

**Fig. 1:**
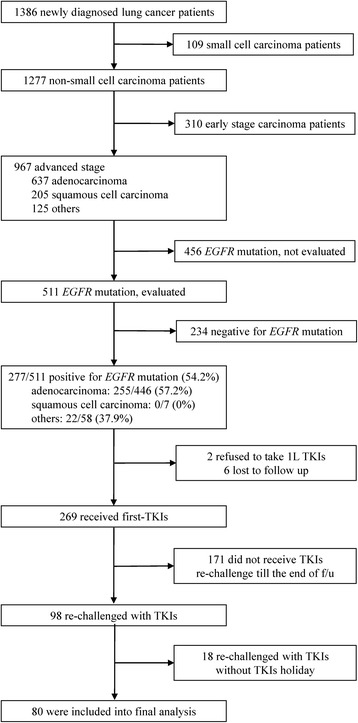
Inclusion, screening, and assignment of patients into groups

Between December 2010 and December 2013 1386 lung cancer cases were diagnosed. Of these, 269 patients had a positive EGFR mutation status and were treated with first-line EGFR-TKIs, and 80 patients were readministered TKIs with at least one cycle intercalated cytotoxic agent (Fig. 1). Lines and regimens of Intercalated chemotherapies were shown in Additional file 1: Table S1. The median follow-up time since readministration was 7.0 months the longest follow-up duration was 20.4 months. At the end of follow-up 78.8 % (63/80) patients showed disease progression under TKI readministration and 36.3 % (29/80) patients were alive. Baseline values and trends of hematological parameters were available for 78 and 77 patients, respectively. To evaluate baseline values and trends of NLR and LMR, using ROC curve analysis, we determined that the best cut-off values were 5.2, 1.1, 2.5, and 0.5, respectively.

### Impact of clinical factors on overall survival of TKI readministration

**Fig. 2:**
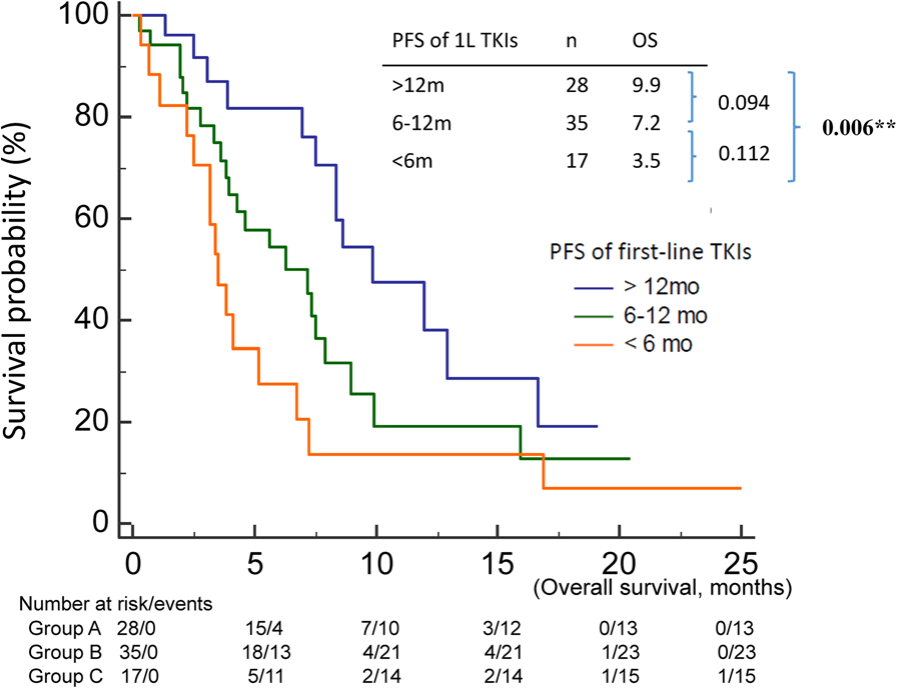
Overall survival since the readministration of tyrosine kinase inhibitors of patients with short (<6 months), intermediate (6–12 months), and long (>12 months) progression free survival of first-line tyrosine kinase inhibitors

**Fig. 3:**
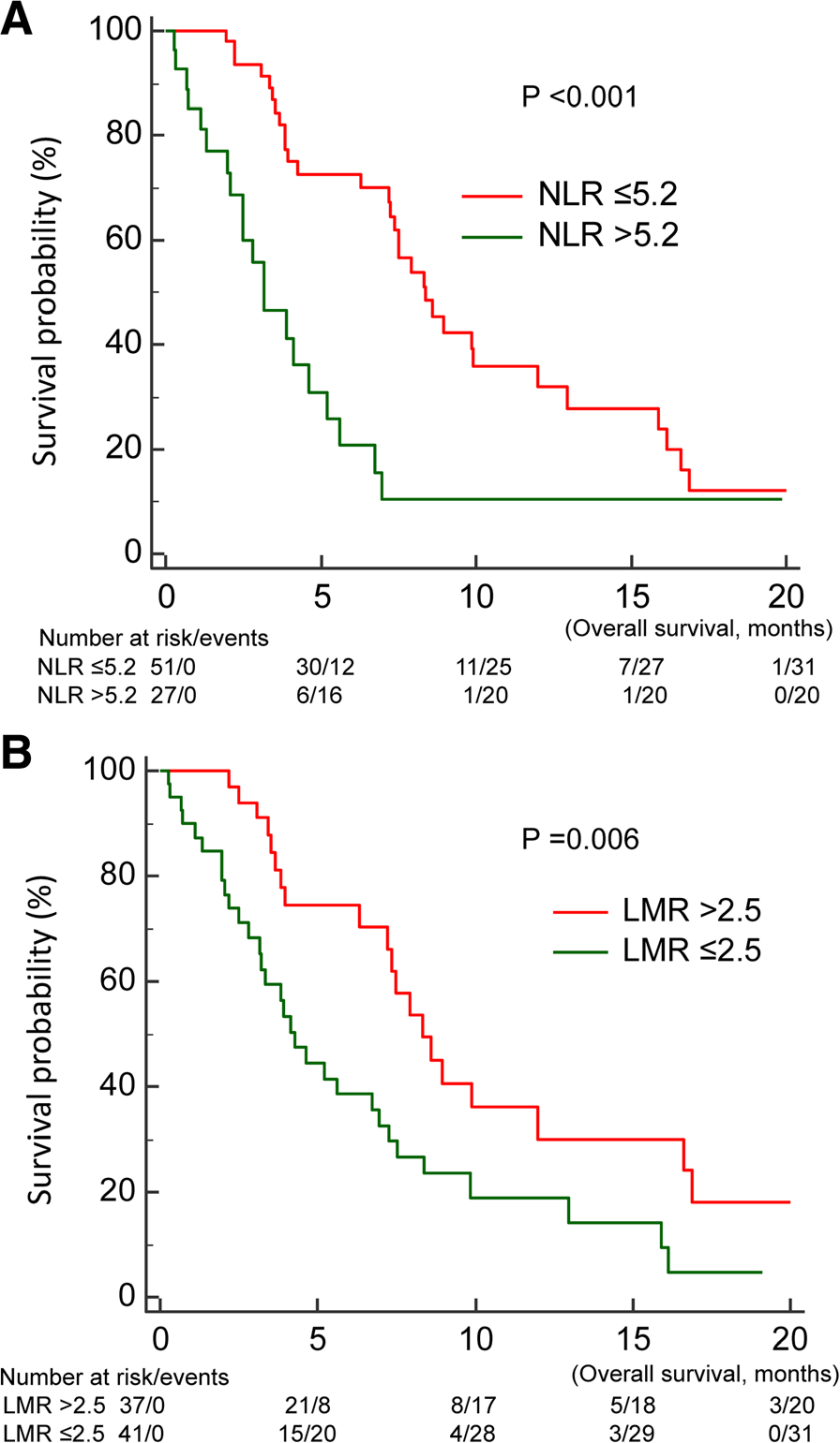
Influence of baseline proinflammatory markers on overall survival (OS) of patients who were readministered with tyrosine kinase inhibitors (**a**) OS between patients with high and low baseline neutrophil-to-lymphocyte ratio (NLR); (**b**) OS between patients with high and low lymphocyte-to-monocyte ratio (LMR)

**Fig. 4:**
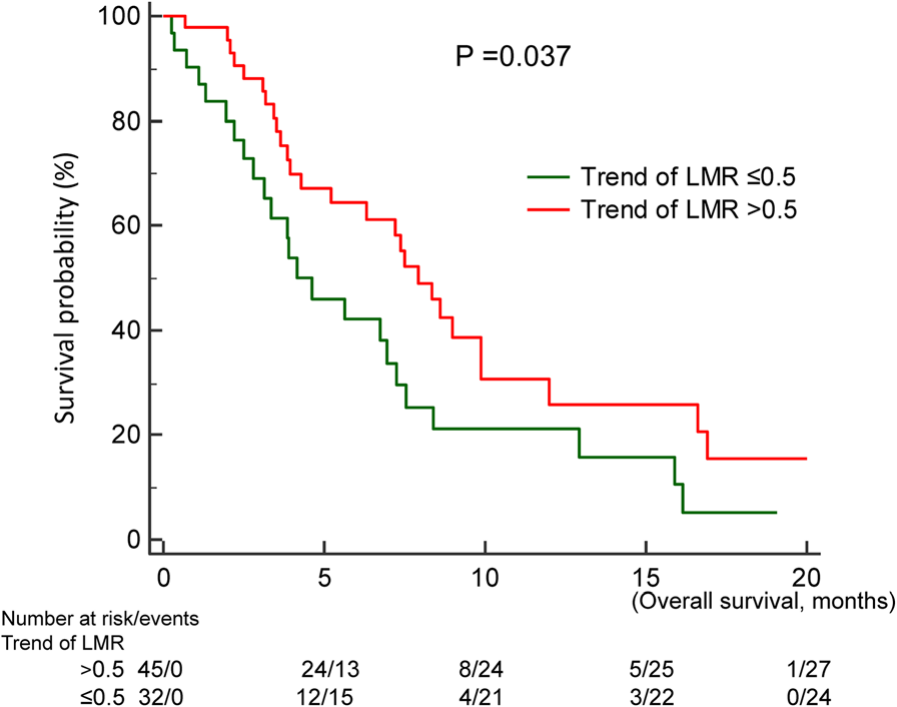
Influence of trends of lymphocyte-to-monocyte ratio (LMR) on overall survival (OS) of patients who were readministered with tyrosine kinase inhibitors OS between patients with high and low trend of LMR

Clinical factors found to be significant in the univariable analysis for poor OS since TKI readministration included shorter PFS of first-line TKI (*p* = 0.020) (Fig. 2) high baseline NLR (*p* < 0.001) (Fig. 3a), low baseline LMR (*p* = 0.006B), and low trend of LMR (*p* = 0.037) (Fig. 4) (Table 1).

**Table 1:** Clinical factors and systemic inflammatory status of patients receiving EGFR-TKI readministration

	Univariable analyses			Multivariable analyses		
Characteristics	N (%)	OS	p	Hazard ratio	95 % CI	*P* value
Length of EGFR-TKI holiday			0.235			
< 3	16 (20.0)	3.8				
3–6	25 (31.3)	6.7				
> 6	39 (48.8)	8.4				
PFS of first-line EGFR-TKI			0.020			<0.001
< 6	17 (35.0)	3.5		4.970	2.170–11.382	
6–12	35 (43.8)	7.2		1.818	0.899–3.678	
> 12	28 (21.3)	9.9		1		
Changes in the EGFR-TKI regimen			0.474			
Yes	75 (93.8)	7.2				
No	5 (6.2)	8.4				
Type of EGFR-TKI readministrated			0.934			
1^st^ generation	71 (88.8)	7.0				
2^nd^ generation	9 (11.2)	7.4				
Baseline NLR			<0.001			0.037
> 5.2	27 (34.6)	3.2		2.352	1.052–5.256	
≤ 5.2	51 (65.4)	8.4		1		
Trend of NLR			0.129			
> 110 %	44 (57.1)	4.3				
≤ 110 %	33 (42.9)	8.4				
Baseline LMR			0.006			0.632
> 2.5	37 (46.8)	8.3		1		
≤ 2.5	41 (53.2)	4.2		1.197	0.574–2.497	
Trend of LMR			0.037			0.004
> 50 %	45 (58.4)	7.9		1		
≤ 50 %	32 (41.6)	4.1		2.651	1.374–5.118	

*Abbreviations: CI* confidential interval, *EGFR* epidermal growth factor receptor, *LMR* lymphocyte to monocyte ratio, *NLR* neutrophil to lymphocyte ratio, *OS* overall survival, *PFS* progression-free survival, *TKI* tyrosine kinase inhibitor

Length of TKI holiday changes in the TKI regimen, and first or second generation TKIs when TKI readministration, and trend of NLR did not significantly influence OS. In the multivariable analysis, independent prognostic factors for shorter OS were shorter first-line TKI PFS (*p* < 0.001), high baseline NLR (*p* = 0.037), and low trend of LMR (*p* = 0.004) (Table 1).

## Discussion

Our retrospective observational study found that baseline NLR and trend of LMR as well as PFS of first-line EGFR-TKI treatment were prognostic factors in patients receiving TKI readministration. NLR was previously found to have a prognostic effect in different types of cancer like ovarian cancer, breast cancer, pancreatic cancer, and colorectal cancer, as well as in advanced NSCLC patients treated with first-line platinum-based chemotherapy [16–21]. LMR was found to be a prognostic factor in small cell lung cancer [22], in early-stage NSCLC patients post operation [23], in advanced lung cancer treated with cytotoxic chemotherapies [24], and in EGFR-mutant lung cancer patients treated with first-line EGFR-TKIs [14]. Several possible mechanisms may explain the prognostic effect of these pro-inflammatory markers. First, neutrophils release several pro-angiogenic factors and promote angiogenesis, which is essential for tumor progression. Second, lymphocytes play a pivotal role in tumor cell eradication [25], and tumor-associated macrophages promote tumor progression through remodeling of the tumor extracellular matrix [26, 27]. Based on the above pathophysiology, patients with high NLR and low LMR tend to have tumor progression and fewer T cells available for cancer cell eradication.

Previous studies have reported conflicting results regarding the influence of PFS of previous EGFR-TKI on the efficacy of TKI readministration. In one study that included all patients without TKI holidays longer PFS of previous TKI treatment paradoxically shortened the PFS of TKI readministration [11]. Another study in which 52 % of patients with a TKI holiday before TKI rechallenge revealed that PFS of previous TKI treatment was not related with the efficacy of TKI readministration [12]. By excluding patients without having TKIs holidays, our study revealed that patients with a longer PFS of previous TKI treatment have a longer OS of TKI readministration. In the first study, the authors speculated that in patients who received previous therapy for less than 12 months, the tumor may not yet have acquired the 790 M mutation. However, this concept was not supported by subsequent studies [28]. We speculated that when the disease progresses after the first TKI therapy, tumors have a dominant part of TKI-resistant clones and a minor part of TKI-sensitive clones.

After the TKI holidays and owing to intercalation with cytotoxic chemotherapies tumor redistribution occurred, which lead to TKI-sensitive clones increasing, and TKI-resistant clones decreasing. This redistribution was due to higher sensitivity to cytotoxic chemotherapies in TKI-resistant clones than that in TKI-sensitive clones. After tumor redistribution by the intercalated chemotherapies, tumor characteristics were more similar to those of TKI-naïve tumors than to TKI-resistant tumors.

This can explain at least partly, why PFS of previous TKIs has opposite influences in patients with or without TKI holidays. However, this concept should be proved with further studies.

Though several studies have reported on how clinical factors affect the efficacies of TKI readministration [10–12] patient heterogeneity is a confounding factor that cannot be neglected. One study included more than 70 % of patients without TKI holidays, whereas two other studies included 32 and 50 % patients with wild type EGFR mutation, respectively. We only included EGFR-mutant NSCLC patients receiving first-line EGFR-TKIs and at least one intercalated chemotherapy agent to decrease these confounding factors. To the best of our knowledge, this is the first study demonstrating that baseline NLR and trend of LMR are prognostic factors in patients receiving EGFR-TKI readministration. As a study aimed at patients receiving third and later line therapies, the number of patients is not small.

Our study had several limitations. First, data regarding the amount and pattern of inflammatory cell infiltration as well as the amount of tumor programmed death-ligand 1 expression in tumors were not available, which could have provided us further information about the immune condition in the tumor microenvironment [29]. Further studies are required to determine whether immunotherapy or anti-angiogenesis agents could prolong survival in those who were speculated to have poor prognosis to TKI readministration. Finally, our study was a retrospective study a prospective trial is needed to validate these results.

## Conclusion

Longer PFS of first-line TKIs, low baseline NLR, and high trend of LMR were good prognostic factors in EGFR-mutant NSCLC patients receiving TKI readministration.

## Additional file

Additional file 1: Lines and regimens of Intercalated chemotherapies. (DOCX 12 kb)

## Abbreviations

EGFR: Epidermal growth factor receptor
LMR: Lymphocyte-to-monocyte ratio
NLR: Neutrophil-to-lymphocyte ratio
NSCLC: Non-small cell lung cancer
OS: Overall survival
PFS: Progression free survival
ROC: Receiver operating characteristic
TKI: Tyrosine kinase inhibitor
