# Supplementary material for: Impact of clinical parameters and systemic inflammatory status on epidermal growth factor receptor-mutant non-small cell lung cancer patients readministration with epidermal growth factor receptor tyrosine kinase inhibitors
Source: BMC Cancer. 2016 Nov 8;16:868. doi: 10.1186/s12885-016-2917-6 (PMC5100346; doi:10.1186/s12885-016-2917-6)
Supplement: Additional file 1: — Lines and regimens of Intercalated chemotherapies. (DOCX 12 kb) [file 12885_2016_2917_MOESM1_ESM.docx]

Supplement table 1

| *2^nd^ line chemotherapy* | *N=61* |
| --- | --- |
| *Platinum* + Pemetrexed*  *platinum + Gemcitabine*  *platinum + Docetaxel*  *platinum + Vinorelbine*  *Pemetrexed*  *Gemcitabine*  *Docetaxel*  *Vinorelbine* | *45 (73.8%)*  *8(13.1%)*  *9 (14.8%)*  *2 (3.3%)*  *6 (9.8%)*  *1 (1.6%)*  *2 (3.3%)*  *7 (11.5%)* |
| *3rd line chemotherapy* | *N=19* |
| *Pemetrexed*  *Docetaxel*  *Paclitaxel*  *Vinorelbine* | *4(21.1%)*  *12(63.2%)*  *1(5.3%)*  *2 (10.5%)* |
| *4^th^ line Chemotherapy* | *N=1* |
| *Vinorelbine* | *1(100%)* |
